# Sepsis: New horizons

**DOI:** 10.4103/0972-5229.63026

**Published:** 2010

**Authors:** Subhash Todi

**Affiliations:** **From:** Department of Critical Care & Internal Medicine, AMRI, Kolkata, India

Sepsis is a major cause of morbidity and mortality in intensive care units (ICU) worldwide. In a survey conducted in eastern India, severe sepsis (sepsis with organ dysfunction) constituted 17% of all admissions to the ICU. This cohort carried a very high mortality of 45%, much higher than a similar cohort from the west. It is appropriate that the Indian Journal of Critical Care Medicine (IJCCM) has decided to bring out an issue with sepsis as the theme, as it will help in increasing awareness of this problem among the readers.

Sepsis is a heterogeneous disorder, with varied presentations. Conceptually it may be defined as a host response to an infection. Attempts have been made to arrive at an agreeable definition, but this can only help in selecting some patients among this heterogeneous population, for trial purposes. At present we do not have a highly sensitive or specific tool for detecting sepsis at the bedside, which leads to an overuse of antibiotics, 50% of which are inappropriate, with all their attendant consequences. Among the general population and also among the physician community, awareness of sepsis as a disease entity, like cancer, myocardial infarction, and cerebrovascular accident, is very poor. There is hardly any public demand for research in this field, as opposed to say HIV, leading to a limited progress of science in this field. It is the critical care community, which has to deal with this problem on a daily basis and it is up to us to disseminate the awareness of this problem among the public and our colleagues.

The thematic issue on sepsis has three original articles and it is heartening to see quality research coming out of India and critical care practitioners should take the lead in this direction. The article on microalbuminura by Dr. Basu et al., explores an important aspect of the use of biomarkers in sepsis. As we know, the clinical features of sepsis are nonspecific and use of biomarkers adds to the diagnostic acumen of the clinician. Microalbuminuria, similar to other biomarkers like procalcitonin, has a high negative predictive value, but low positive predictive value. Nonetheless, its easy availability, rapid turnover time, and inexpensiveness, is very welcome in the context of the generaisation of its use in our country. Unfortunately, research like this does not get adequate press coverage, and we are still reluctant to accept findings from indigenous research and adapt it in our practice, due to various reasons. Another original article by Dr. Jain *et al*., also addresses an important issue in the management of sepsis. After volume resuscitation, vasopressors are the most widely used drugs for hemodynamic stabilization in sepsis patients. Norepinephrine and Dopamine are the two most widely used vasopressors, but they are expensive and an alternative such as phenylephrine was compared to norepinephrine in this study. However, one has to go beyond global hemodynamics and look at regional perfusion with these agents, as each has a differential effect on organ perfusion. It is sufficient to say that this is a welcome study, which highlights a cheaper alternative to standard vasopressors in sepsis. It is imperative that a clinical research such as this, in a resource-constrained country like ours, be included in the cost-effective analysis, so that the readers get a clearer perspective of its applicability. One should probably add Cost Needed to Treat (CNT) along with other summary statistics like Numbers Needed to Treat (NNT). Similar to our annual congress, our journal is also becoming truly international, with overseas authors contributing to it. The original article by Sakamato *et al.* from Japan highlights an important modality of adjunctive therapy in sepsis. Endotoxin neutralization has been tried with disappointment in various sepsis trials. This novel approach looks at the extracorporeal removal of endotoxin. Interestingly, the agent used for adsorbing endotoxin in this model (polymyxin), is widely used in Indian ICUs as a therapy for multidrug-resistant, gram-negative sepsis. Few centers in India also have experience with this technique and will hopefully publish their data.

This thematic issue also has two review articles. One by Divatia *et al.,* our ex-president, which gives an overview of sepsis. It also highlights recent trials on sepsis and application of surviving sepsis guidelines in our country. This article highlights an important issue of collaborative research. As we have seen with Australian, New Zealand, Canadian, and recently Chinese Critical Care trial groups, mega trials can be conducted in a short time, to arrive at a definite conclusion. In the Asia Pacific Region there is tremendous scope for a similar collaboration and this should be explored actively, and ISCCM should play the leading role in this. Another review article by Majumdar *et al.,* a busy nephrologist, with an interest in critical care, summarizes the important role this organ plays in the outcome of sepsis patients. It also explains the pathophysiology, recent definition, and recent advances in the field of acute kidney injury in sepsis. This contribution also highlights the important contribution our colleagues from other disciplines can make, and reflects the multidisciplinary nature of our speciality. Moreover, I think the success of critical care as a discipline in our country needs acceptance of our role as specialists, by our colleagues from other specialities.

This issue also brings out an important supplement on the Guidelines on Pediatric Sepsis, by Khilnani and Singhi *et al.,* which is adapted to a resource-constrained country like ours. This article is a fine example of collaborative work and will go a long way in standardizing the delivery of care to septic children. I commend the group for their efforts.

With increasingly busy time schedules for all of us, it is becoming difficult to keep up with the recent literature. A summary article on the research published in peer review journals gives an opportunity to the readers to keep abreast of the recent developments in their field, in a short time. All major articles on sepsis were sought in the ‘Top stories of 2009,’ and the ones with relevance to our practice were selected. These summary articles will also help young researchers to select relevant research topics, and explore in depth, their topics of interest. These summary articles are also of use to postgraduates, to be up-to-date with the recent research in this field.

I would like to thank Dr. Sandhya Talekar, our outgoing editor for her tireless work, and welcome Dr. Shirish Prayag our incoming editor. I congratulate them in their effort to get our journal indexed in PubMed.

I would also like to thank Dr. Jigi Divatia our outgoing president for his contribution, especially in launching the collaborative study in India (INDCAP, MOSAIC, AKI_EPI), and welcome our new president Dr. Rajesh Chawla, who will undoubtedly steer us to new heights in the coming years.

